# Resistance of *in natura* and torrefied wood chips to xylophage fungi

**DOI:** 10.1038/s41598-019-47398-9

**Published:** 2019-07-30

**Authors:** Vinícius Resende de Castro, Matheus Perdigão de Castro Freitas, Antônio José Vinha Zanuncio, José Cola Zanuncio, Paula Gabriella Surdi, Angélica de Cássia Oliveira Carneiro, Benedito Rocha Vital

**Affiliations:** 1 Departamento de Engenharia Florestal, Universidade Federal de Viçosa, Viçosa, 36570-900 Brazil; 2 Departamento de Entomologia/BIOAGRO, Universidade Federal de Viçosa, Viçosa, 36570-900 Brazil; 3 Instituto de Ciências Agrárias, Universidade Federal de Uberlândia, Monte Carmelo, 38500-000 Brazil

**Keywords:** Plant sciences, Solid biofuels

## Abstract

The diversity of fungi allows for their colonisation in different environments, including wood destined for power generation, with an ability to degrade or hinder its use. Torrefaction or pre-carbonisation, a low oxygenation heat treatment with temperatures between 200 and 300 °C, accumulates carbon and lignin, decreases hygroscopicity, increases energy efficiency and reduces the wood attractiveness to xylophagous microorganisms. This work aimed to study the resistance of *Eucalyptus urophylla* wood chips, submitted to torrefaction temperatures of 180, 220 and 260 °C for 20 minutes, to xylophagous fungi, according to the ASTM D-2017 method (2005). The white rot fungi *Phanerochaete chrysosporium*, *Pleurotus ostreatus* and *Trametes versicolor* and the brown rot fungus *Gloeophyllum trabeum* were used. After 12 weeks of exposure, the mass losses of wood samples in natura and torrified at 180 °C attacked by *Pleurotus ostreatus* and *Trametes versicolor* was higher. Torrefaction increased the resistance to fungi; the treatment at 260 °C was the most efficient with lower mass losses caused by fungi attacks and, consequently, greater resistance to the fungi tested.

## Introduction

Wood is subject to deterioration by xylophagous microorganisms, including fungi, which secrete enzymes that degrade polymers, transforming them into smaller molecules^1–3^. The great diversity of fungi allows them to colonise diverse environments, such as air, soil and trees. The rotting, mould or stain fungi can totally decompose wood or only mage its surface^4,5^.

Mould and stain fungi are usually the first to colonise the trunk of freshly cut trees and are mainly responsible for patches and changes in the wood surface. Rot fungi, such as basidiomycetes, cause white and brown rot^6^ and can degrade the cell wall, altering the physical and mechanical properties of the wood^7,8^. White rot fungi decompose cellulose, hemicellulose and lignin, leaving the wood clear^9,10^, whereas brown rot fungi degrade cellulose and the cell wall hemicelluloses, altering the mechanical resistance of the material without affecting the lignin, which gives the wood a dark brown appearance^11^.

Fungi initially colonise the wood core with their hyphae, forming a network that is not always visible to the naked eye, filling the cell lumen and passing through the cell wall from one cell to another^8,12^. These fungi can destroy the structure of the middle lamella and change the chemical composition, reducing the wood mass and mechanical resistance^13^. Wood used for power generation is chipped, usually in the field or at the factory. These chips are deposited on patios and stored for approximately 90 days to reduce the wood moisture, when fungus colonisation can modify the wood and reducing its energy potential.

Torrefaction, a heat treatment at controlled temperatures and low oxygenation, can minimise the impacts of rot fungus by increasing the carbon and lignin in the wood^14–16^. This treatment increases the energy density and reduces the hygroscopicity and attractiveness of the material to decomposing microorganisms^17–19^.

The objective of this study was to evaluate the resistance of torrefied *Eucalyptus urophylla* chips to biological deterioration by xylophage fungi.

## Results and Discussion

The equilibrium moisture content (UEH) of the treatments ranged from 5.08 to 12.49% (Table 1), with decreasing values as the torrefaction temperature increased, with lower values from 220 °C. The equilibrium moisture content was 55.96 and 59.33% lower with torrefaction at 220 and 260 °C, respectively, than in the control (*in natura*). The reduction of wood hygroscopicity is due to the faster degradation of cellulose and hemicellulose at lower temperatures, compared to lignin^20^, which reduces the water adsorption capacity^21,22^ and the equilibrium moisture content^6,23^. This reduction is desirable for energy purposes, since a lower energy quantity will be used to evaporate the water contained in the chips^24,25^.

**Table 1:** Equilibrium moisture content (EMC) and chemical composition of the *Eucalyptus urophylla* chips with or without torrefaction.

Properties	Torrefaction temperature			
	In natura	180 °C	220 °C	260 °C
EMC (%)	12.49 ± 1.96 a	9.11 ± 0.61 b	5.50 ± 0.15 c	5.08 ± 0.21 c
Holocelluloses (%)	69.21 ± 1.00 a	70.17 ± 0.51 a	60.48 ± 1.43 b	45.64 ± 0.75 c
Total lignin (%)	26.87 ± 0.91 b	25.37 ± 0.72 b	32.26 ± 1.20 b	47.54 ± 0.16 a
Extratives (%)	3.63 ± 0.05 d	4.24 ± 0.10 c	7.00 ± 0.15 a	6.47 ± 0.27 b
Ashes (%)	0.29 ± 0.05 b	0.22 ± 0.03 b	0.28 ± 0.0,03 b	0.35 ± 0.02 a

Means followed by the same letter, per line, do not differ (Tukey p > 0.05).

Holocellulose was the main wood chemical component that was degraded at high temperatures, with a reduction from 69.21 to 45.64%, compared to the control and a temperature of 260 °C, respectively (Table 1). This compound represents the sum of the cellulose and hemicellulose content, with the latter being degraded at lower temperatures, between 220 and 315 °C, compared to the other primary constituents (cellulose and lignin)^26,27^. The reduction in holocellulose content is mainly due to hemicellulose degradation, justifying the differences between treatments above 220 °C^17^. The total lignin content in the treatments submitted to torrefaction increased by 87,38% compared to the *in natura* and that at 260 °C ones (Table 1). Lignin degradation begins at 160 °C, but traces of this structure can be found at 900 °C^17^. Lignin is the thermally stable chemical compound of the cell wall and it is desired for energy purposes. Therefore, it increases the calorific value of the material and the gravimetric yield in torrefaction and carbonisation^4,26^.

The total extractive content increased with the torrefaction temperature, being greater at 220 °C (Table 1). Hemicellulose degradation generates compounds that remain in the biomass as molecules with fragile fibre connections, which are removed by alcohol/toluene; this increases its extractive content in treatments up to 220 °C^28,29^. The polar extractives degrade in the 130 to 250 °C range, and together with hemicelluloses that volatilise at high temperatures, this explains the decrease in extractive content in the treatment at 260 °C. An increase in the extractive content in treatments up to 220 °C was also reported for grasses^30^ and coniferous woods^29^.

The ash content increased with the torrefaction temperature, being 20.7% higher in the torrified material at 260 °C than in the *in natura* treatment (Table 1). This may be caused by the organic biomass, such as the degradation and loss of the hemicelluloses, but the ash content varies between plant species and clones^31,32^. Materials for heat generation must have a low ash content to reduce the potential energy losses and the corrosion of the equipment used for the biomass combustion^26,33^.

Torrefaction increased the resistance of the material to fungi deterioration and reduced the mass loss as the temperature increased (Table 2). The wood degradation (mass losses) is related to the degradation and chemical alteration of carbohydrates, preventing fungi from feeding on wood^34^. These results corroborate with the lower mass losses of softwoods and hardwoods by fungi, after thermoretification at high temperatures with longer exposure periods^34,35^. Torrefaction increased the wood chip resistance to degradation by the xylophagous fungi *Trametes* sp. and *P. ostreatus*, with lower mass losses at 260 °C (Table 2). The 1.62% mass loss of non-torrefied wood submitted to *T. versicolor* was similar to that of *Eucalyptus tereticornis* with this fungus, which was 2.06% after 12 weeks of exposure^36^.

**Table 2:** Mass losses (%) of pine wood and *Eucalyptus urophylla* chips in natura and torrified to the attack of xylophagous fungi.

Fungus	Torrefaction temperature				
	Pinus	In natura	180 °C	220 °C	260 °C
*Gloeophyllum trabeum*	2.05 A	0.91 Bb	1.01 Ba	0.92 Ba	0.37 Ba
*Phanerochaete chrysosporium*	6.95 A	1.19 Bb	1.22 Ba	−0.13 Bb	−0.32 Ba
*Pleurotus ostreatus*	5.84 A	2.78 Ba	1.85 BCa	0.00 CDb	−0.51 Ea
*Trametes versicolor*	5.56 A	1.62 Bab	0.86 BCa	0.60 BCa	−0.15 Da

Means followed by the same capital letter, per line, or the same lowercase letter, per line, conferir isto do not differ by Tukey test (p > 0.05).

**Figure 1:**
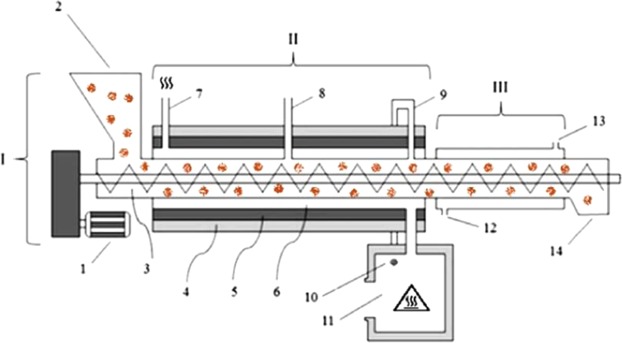
*Trametes* sp fungus hyphae fixed to the wood chips at 260 °C.

Torrefaction reduced the holocellulose contents and increased the extractives and lignin, justifying the higher resistance of the wood chips treated at 260 °C to *T. versicolor* and *P. ostreatus* fungi, which preferentially degrades cellulose and hemicellulose^6^. Exposure to high temperatures chemically modifies the wood due to hemicellulose degradation, which is a source of fungi food, generating extractives with fungicide action^37^. This alters the material hygroscopicity^38–40^ and leaves it with an acidic pH, hindering the fungi development^6,18^. The wood chip mass with *G. trabeum* and *P. chrysosporium* was similar between treatments, including that of the torrified ones (Table 2). Substances such as extractives make wood more resistant to deterioration by xylophages and the torrefaction of the material generates/accumulates extractives that minimise fungi damage. Extractives have chelating agents, capable of forming complexes with metals, that protect the wood and, if more concentrated, they can be natural preservatives (fungicides and insecticides)^37,41^. The mass of the torrefied wood chips at 260 °C increased with most fungi (Table 2), may be due to fungi colonisation without the degradation of the chips, with an increase in the mass of their hyphae, which were strongly fixed in the material and not, completely, removed during the chip cleaning process (Fig. 1).

The increase in the wood chip resistance to degradation by white rot fungi (Table 2) is due to the higher lignin contents of this material, caused by torrefaction. Moreover, these fungi decompose holocelulose and lignin, as reported for the wood of *Fagus sylvatica*^4^ and *Hevea brasiliensis*^42^. Degradation of *in natura* wood chips was higher with *P. ostreatus* white rot fungus and lower with *Gloeophyllum trabeum*, which is due to the lower lignin contents in hardwoods, favouring the development of white rot fungi (*P. ostreatus*) compared to brown rot fungi (*G. trabeum*). The mass losses of torrified *Eucalyptus urophylla* samples varied from 0.37 to 1.01% with the fungus *Gloeophyllum trabeum*, similar to those reported (from 1.29 to 1.65%) for *Corymbia torelliana* and *Eucalyptus grandis* x *Eucalyptus urophylla* for this brown rot fungus^43^.

Wood chip degradation at 220 °C by the *G. trabeum* and *Trametes* sp. fungi differed from the treatments with lower torrefaction temperatures due to variations in the nutritional requirements of each xylophagous fungus^6^. Brown rot fungi release enzymes that diffuse from the cell lumen, where their hyphae degrade carbohydrates in the secondary cell wall layer S2, S1 and S3, in sequence. On the other hand, white rot fungi gradually attack the cell wall constituents from the lumen outward, first attacking the S3 layer, then progressively attacking the other layers. This explains the correlation between the quantity and characteristics of the carbohydrates and the cell wall resistance to deterioration^6,37^.

The decay susceptibility indices in the treatments with and without torrefaction were lower than 100, indicating that the studied wood was more resistant than the reference one (Table 3). A low decay susceptibility index indicates greater resistance to degradation because the chemical composition of the substrate is not attractive to fungi due to torrefaction. The changes in the chemical composition of *Eucalyptus nitens*, *Eucalyptus globulus* and *Alnus incana* make wood less susceptible to the *Trametes versicolor* attack^44,45^.

**Table 3:** Decay susceptibility index (%) of *Eucalyptus urophylla* chips with to xylophagous fungi with or without torrefaction.

Fungus	Torrefaction temperature			
	In natura	180 °C	220 °C	260 °C
*Gloeophyllum trabeum*	44.39 Aa	49.28 Aa	44.90 Aa	18.12 Aa
*Phanerochaete chrysosporium*	17.12 Ab	17.55 Ab	−1.87 Ab	−4.60 Ab
*Pleurotus ostreatus*	47.57 Aa	31.65 ABab	0.02 BCb	−8.76 Db
*Trametes versicolor*	29.08 Aab	15.47 ABb	10.77 ABb	−2.73 Cb

Means followed by the same capital letter, per line, or by the same lowercase letter, per column, do not differ by Tukey test (p > 0.05).

Torrefaction reduced the decay susceptibility index of wood samples to *P. ostreatus* and *Trametes* sp. due to the decrease in holocellulose content, mainly at the higher temperatures, 220 and 260 °C. On the other hand, high sugar and starch levels stored in the cells increase wood susceptibility to rot fungus^6^. Wood chip torrefaction increases the energy potential of the product and the resistance to deterioration by xylophagous fungi, as found for *Myracrodruon urundeuva* or *Schinopsis brasiliensis*, with higher mass losses of 0.99 and 1.35%, respectively^46^ and *Astronium* sp., with a mass loss of 1.97% by the fungus *Gloeophyllum trabeum*^47^.

Torrefaction reduced the wood equilibrium moisture content and changed its chemical composition. An increase in temperature decreased the holocellulose content and increased the contents of ash and lignin; the extractive content increased in the treatments up to 220 °C and decreased at 260 °C. Torrefied *Eucalyptus urophylla* wood chips are resistant to *Gloeophyllum trabeum, Phanerochaete chrysosporium*, *Pleurotus ostreatus* and *Trametes versicolor* fungi according to ASTM D-2017^48^. However, the degradation of the *in natura Eucalyptus urophylla* chips was higher with *Pleurotus ostreatus* and lower with *Gloeophyllum trabeum*. Torrefaction increased the wood chip resistance to the xylophagous fungi, with the treatment at 260 °C being the most efficient for all evaluated fungi.

## Methods

*Eucalyptus urophylla* wood chips *in natura* and torrified were submitted to xylophage fungi in an accelerated rotting test, according to the American Society for Testing and Materials - ASTM D-2017^48^.

### Torrefaction process

The wood chips of seven-year-old *E. urophylla* were used. These chips were sieved; those that passed through the 31.5 mm sieve, but were retained in the 16 mm sieve, were used in the experiment. The selected chips were oven dried at 103 ± 2 °C to reach 0% moisture and torrified for 20 minutes at 80, 220 and 260 °C.

**Figure 2:**
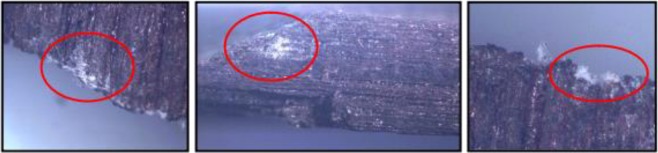
Lateral layout of a prototype screw reactor developed by a Brazilian university for thermal treatment of lignocellulosic biomass, where: I- transport system; II- heating system; III- cooling system; 1- motor; 2- input biomass; 3- worm-screw; 4- insulating layer; 5- refractory layer; 6- flow of heating gas; 7- heating gas output; 8- first “chimney”; 9- second “chimney”; 10- connection “chimney” with the burner; 11- connecting burner; 12- water supply; 13- water outlet; 14- exit of torrefied biomass.

Torrefaction was performed in an endless screw reactor, developed in the Panels and Wood Energy Laboratory (LAPEM/UFV)^15^. The metal prototype of this equipment was a semicontinuous screw reactor, which reuses the volatile gases in the heating system (Fig. 2).

### Chemical characterization and equilibrium moisture content

The wood equilibrium moisture content of the wood samples was calculated on a dry basis; the wood samples were placed in a climatic chamber at 20 °C and 65% relative humidity until constant mass. Samples were milled with a Standard Wiley knife mill with a 2 mm screen. The material that was sieved with a 40–60 mesh sieve and the retained fraction were used to determine its structural chemical composition, according to the standards of the Technical Association of the Pulp and Paper Industry^49^, such as the wood preparation for the chemical test (T264)^50^; extractive content (T204)^50^; lignin content (T222)^50^; and cellulose and hemicellulose content (T223)^50^. The ash content was determined according to NBR 8112/83^51^.

### Biological assay evaluation

The experiment followed the ASTM standard D-2017^48,52^ procedures. The samples were submitted to the white rot fungi *Phanerochaete chrysosporium*, *Pleurotus ostreatus* and *Trametes versicolor* and to the brown fungus *Gloeophyllum trabeum*. The wood was dried at 103 °C and all equipment, glassware and soil were autoclaved to prevent contamination. Each 600 mL vase was filled with 300 g sterilized clayey soil at a pH of 6.8 and moistened with 83 mL distilled water with a water retention capacity of 35.68%. The samples were submitted to fungi colonisation for 12 weeks, after which they were removed and the final oven dried weight was obtained.

The accelerated test aims to predict potential for fungal biodeterioration in a shorter period. The time is reduced, only 12 weeks, however, the test provides all conditions for optimal growth of the fungus (temperature, humidity, pH and etc). The sample mass losses in the 12-week test is expected to be similar to that of wood attacked by the fungus in the field, even if the colonization demands a greater period. The resistance class and the decay susceptibility index of the material were evaluated according to the initial and final mass of the specimens, as ASTM D-2017^48^ described, in equation 1:

$${\rm{ML}}=(({\rm{Mi}}-{\rm{Mf}})/{\rm{Mi}})\times 100$$

where: ML = sample mass losses; Mf = final sample mass and Mi = initial sample mass; and equation 2

$${\rm{DSI}}=({\rm{MLi}}/{\rm{MLr}})\times 100$$

where: DSI = decay susceptibility index; MLi = mass loss of the sample tested; MLr = mass loss of the reference species (*Pinus* sp.). Pine wood is used as a reference because it has low resistance to fungi attack, being used as comparative parameter.

The wood resistance to fungal attack was classified according to the average mass losses, as follows: 0–10% (Highly resistant); 11–24% (Resistant); 25–44% (Moderately resistant) and 45 or above (Slightly resistant or non-resistant), as suggested by ASTM standard D-2017^48,52^.

### Statistical analysis

The results of the equilibrium moisture content, chemical composition and biological assay in relation to the torrefaction temperature of the *E. urophylla* chips were analysed in a completely randomised design, with four treatments (*in natura* and three torrefaction temperatures) and four fungi with six replications. The means were grouped with a Tukey test (p ≤ 0.05). The statistical analyses were performed with STATISTICA 8.0 software^53^.
